# Coagulation Behavior of Antimony Oxyanions in Water: Influence of pH, Inorganic and Organic Matter on the Physicochemical Characteristics of Iron Precipitates

**DOI:** 10.3390/molecules27051663

**Published:** 2022-03-03

**Authors:** Muhammad Ali Inam, Kang Hoon Lee, Hira Lal Soni, Kashif Hussain Mangi, Abdul Sami Channa, Rizwan Khan, Young Min Wie, Ki Gang Lee

**Affiliations:** 1 Institute of Environmental Sciences and Engineering (IESE), School of Civil and Environmental Engineering (SCEE), National University of Sciences and Technology (NUST), H-12 Campus, Islamabad 44000, Pakistan; ainam@iese.nust.edu.pk; 2 Department of Energy and Environmental Engineering, The Catholic University of Korea, 43 Jibong-ro, Bucheon-si 14662, Korea; 3 Department of Chemical Engineering, Quaid-e-Awam University of Engineering, Science and Technology (QUEST), Nawabshah 67480, Pakistan; soni.hiralal@quest.edu.pk (H.L.S.); kashifmangi@quest.edu.pk (K.H.M.); abdul.sami@quest.edu.pk (A.S.C.); rizwansoomro@quest.edu.pk (R.K.); 4 Department of Materials Engineering, Kyonggi University, Suwon 16227, Korea; supreme98@kyonggi.ac.kr (Y.M.W.); gglee@kyonggi.ac.kr (K.G.L.)

**Keywords:** antimony, coagulation, flocs, organic matter, phosphate, surface properties

## Abstract

The presence of inorganic and organic substances may alter the physicochemical properties of iron (Fe) salt precipitates, thereby stabilizing the antimony (Sb) oxyanions in potable water during the chemical treatment process. Therefore, the present study aimed to examine the surface characteristics, size of Fe flocs and coagulation performance of Sb oxyanions under different aqueous matrices. The results showed that surface properties of Fe flocs significantly varies with pH in both Sb(III, V) suspensions, thereby increasing the mobility of Sb(V) ions in alkaline conditions. The negligible change in surface characteristics of Fe flocs was observed in pure water and Sb(III, V) suspension at pH 7. The key role of Van der Waals forces of attraction as well as hydration force in the aggregation of early formed flocs were found, with greater agglomeration capability at higher more ferric chloride dosage. The higher Sb(V) loading decreased the size of Fe flocs and reversed the surface charge of precipitates, resulting in a significant reduction in Sb(V) removal efficiency. The competitive inhibition effect on Sb(III, V) removal was noticed in the presence of phosphate anions, owing to lowering of ζ-potential values towards more negative trajectory. The presence of hydrophobic organic matter (humic acid) significantly altered the surface characteristics of Fe flocs, thereby affecting the coagulation behavior of Sb in water as compared to the hydrophilic (salicylic acid). Overall, the findings of this research may provide a new insight into the variation in physicochemical characteristics of Fe flocs and Sb removal behavior in the presence of inorganic and organic compounds during the drinking water treatment process.

## 1. Introduction

In the past decade, heavy metal pollution in potable water supplies have received serious attention at a global level because of their potential health risks to humans and the ecosystem. Among many removal technologies, coagulation has been found to be affective in the removal of heavy metals such as antimony (Sb) from drinking water [1,2]. The hydrolysis of iron (Fe) salts plays a crucial role in the removal of Sb and dissolved organic compounds during the chemical coagulation process. The precipitation of Fe^3+^ hydrolysis products mainly involves nucleation and agglomeration phenomena, whereas hydrolysis reactions are very fast followed by nucleation [3,4]. Moreover, the colloidal stability of precipitated Fe floc depends upon several factors of surrounding aqueous environment such as pH, cations, anions and organics compounds. Previous study [5] demonstrated that the primary precipitation process can be understood by classical Derjaguin, Landau, Verwey, and Overbeek (DLVO) theory and Van der Waals forces between the colloids. In the past [6,7], the colloidal and solubility behavior of Fe species were thoroughly investigated under heterogeneous aqueous environment. However, literature seems insufficient to address the influence of zeta potential and size of Fe flocs on the coagulation behavior of Sb oxyanions in water. Therefore, it is crucial to investigate the physicochemical properties of precipitates and simultaneous Sb removal under different aqueous matrices.

The complexity of water chemistry plays an essential role in affecting the electrical properties of primary Fe precipitates and thereby coagulation performance of the system [6,8,9]. For instance, suspension pH has been shown to affect the surface characteristics and solubility behavior of Fe flocs in a few studies [7,9,10]. The recent studies [11,12] have also attempted to optimize the coagulation removal strategy of Sb(V) in the textile wastewater matrix using common Fe coagulants, and found effective removal under acidic pH conditions owing to strong surface interaction between Fe and Sb(V) species. It has also been reported that the agglomeration process of Fe precipitates may be hindered in the presence of inorganic and organic compounds in water [9,13,14]. The organic compounds such as humic acid (HA) and fulvic acid (FA) may form various complexes with Sb and Fe species, thereby affecting the overall systems’ performance [9,14]. In general, the effect of these components were directly related to their basicity and coordinative complexation ability with Fe compared to hydroxide ion [15]. Therefore, the systematic characterization of zeta potential, size and agglomeration of the Fe flocs may provide better understanding about the removal behavior of these complex pollutants from water.

A recent study [7] attempts to explain the influence of pH and HA on pentavalent [Sb(V)] and trivalent [Sb(III)] removal with particular emphasis on the physicochemical properties of Fe precipitates. The significant effect of pH on Sb(V) removal has been evidenced, particularly at alkaline conditions [6,9,16]. The presence of hydrophobic organic compounds such as HA may alter the surface properties of Fe flocs, thereby affecting the overall performance of the coagulation process [7,17]. It has been previously reported that the HA molecules may affect the optimum removal conditions by deviating the point of zero charge of Fe flocs towards charge reversal conditions [14,17]. Earlier studies [2,8,18] also reported the antagonistic effect of anions such as sulfates, phosphates on the removal rate of Sb oxyanions in water. Although several studies provide some insights into the influence of water chemistry parameters on the physicochemical properties of Fe flocs. However, available literature addressing the impact of anions and hydrophobic/hydrophilic organics on the electrical characteristics and aggregation behavior of Fe flocs, as well as their effect on the coagulation of Sb(III, V) oxyanions in water, seems insufficient.

Accordingly, present study systematically examined the surface characteristics of Fe flocs and removal efficiency of Sb(III, V) oxyanions in water during the coagulation process. The influence of pH, ferric chloride (FC) dosages, contaminant loading, phosphate and hydrophobic/hydrophilic organic compounds were also investigated under heterogeneous aqueous environments.

## 2. Results and Discussion

### 2.1. Influence of pH on Fe Precipitates Properties and Sb Coagulation

**Figure 1:**
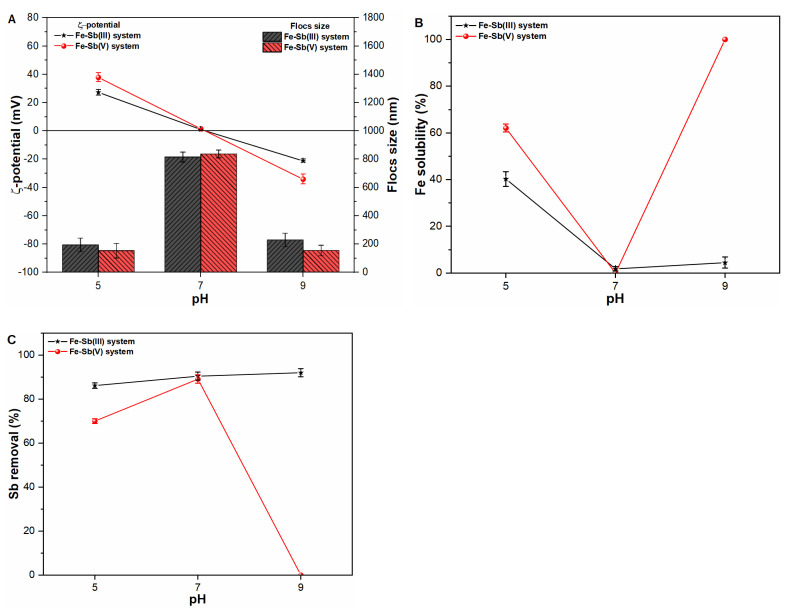
Influence of pH on (**A**) zeta potential (mV) and size (nm) of freshly formed Fe flocs; (**B**) Fe solubility (%) and; (**C**) Sb removal (%) in Sb(III, V) suspensions.

To explore the surface characteristics of coagulated Fe precipitates, the ζ-potential as well as flocs size measurement were conducted at different pH conditions (Figure 1A). It was noteworthy that the isoelectric points (pH_iep_) of Fe precipitates were observed under circumneutral pH environment in both Sb(III, V) suspensions and pure water (Figure 1A and Figure S1A). Such observation may be attributable to the strong interaction of Fe(OH)_2_^+^ with neutral Sb(III) species [Sb(OH)_3_] and negatively charged Sb(V) ions [Sb(OH)_6_^−^] under neutral pH environment (Figure S1C,D). Therefore, the bigger size (around 825 nm) Fe precipitates were formed at pH 7 thus indicating stronger aggregation capacity under those conditions (Figure 1B and Figure S1B). Earlier studies [9,17] also presented similar results, which favored the formation of Fe flocs during conventional coagulation process under neutral pH environment. However, the smaller size Fe flocs in the range of 153–193 nm were formed at acidic and alkaline pH, thus indicating weaker aggregation affinity in those suspensions. Interestingly, the significant decline in Fe flocs formation at alkaline solutions were observed in the presence of Sb(V) ions (Figure 1B). Such behavior may be supported by the interaction of negatively charged Sb(OH)_6_^−^ and Fe(OH)_4_^−^ species (Figure S1C,D). The strong electron donation from Sb(V) complex to surficial Fe atom will enhance electron density, leading to a breakage of the bond at the Fe–O interface [19]. In contrast, Sb(III) exist as Sb(OH)_3_ and strongly interact with Fe(OH)_4_^−^ at alkaline environment, therefore, higher Sb removal was also observed under similar conditions (Figure 1C). In comparison, the Fe precipitates presented excellent coagulation performance at circumneutral pH towards both toxicants. Similarly, earlier studies [9,17,20,21] also reported stronger removal capacity at neutral pH, while working on heavy metals removal from water via Fe-based coagulants.

The results of the pH experiments confirmed that pH has a significant impact on the physicochemical properties and solubility behavior of Fe precipitates. Considering pH 7 as favorable for Fe flocs formation as well as Sb(III, V) removal, as demonstrated here, subsequent coagulation experiments only focused on circumneutral pH conditions.

### 2.2. Influence of FC Dosages on Fe Precipitate Properties and Sb Coagulation

**Figure 2:**
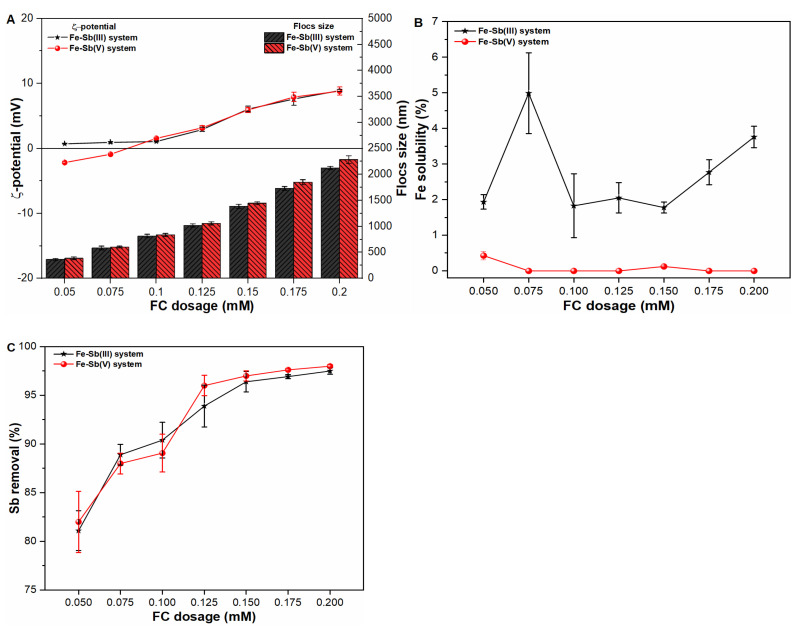
Influence of FC dosages on (**A**) zeta potential (mV) and size (nm) of freshly formed Fe flocs; (**B**) Fe solubility (%) and; (**C**) Sb removal (%) in Sb(III, V) suspensions.

To further explore the physicochemical properties of coagulated Fe, experiments were conducted under various FC coagulant dosages in pure water as well as Sb(III, V) suspensions (Figure 2A and Figure S2A). The results indicated slight variation in ζ-potential values in the range of −2.161 mV to −8.915 mV in all studied waters; however, Fe flocs size was identified as a function of applied FC dosages. The larger Fe flocs size at higher FC dosage might be attributable to the greater agglomeration capacity as a result of greater quantity of available Fe species. The maximum Fe floc size during early stage formation was observed to be 2125.506 nm and 2285.643 nm in Sb(III) and Sb(V) suspensions, respectively. The relatively greater size of Fe flocs in Sb(V) suspension may be ascribed to the stronger interaction of Fe(OH)_2_^+^ and Sb(OH)_6_^−^ as a result of charge neutralization, hence encouraging the flocculation process (Figure S1C,D). Similar to our results, previous study also presented greater diameters of early formed precipitates under the application of aluminum-based coagulant in water [22]. In accordance, a negligible influence of coagulant dosage on Fe solubility behavior was observed in Sb(III, V) suspensions (Figure 2B). As shown in Figure 2C, the increase in Sb(III, V) removal efficiency was observed upon increasing applied FC dosages. Such removal trend indicates that increasing FC dosage resulted in increase in available Fe surface sites thus enhancing the coagulation efficiency of the system. Similarly, earlier studies [10,20] also presented higher Sb removal trend at higher coagulant dosages. In general, considering the greater coagulation performance and negligible residual Fe concentration in treated water, as well as the low toxicity and biocompatibility of Fe coagulant make it suitable for remediating Sb contaminants from drinking water.

### 2.3. Aggregation Behavior of Fe Precipitates

**Figure 3:**
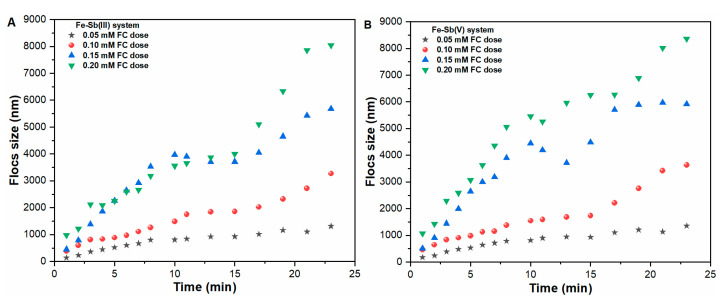
Aggregation of Fe precipitates in (**A**) Sb(III), (**B**) Sb(V) suspensions as a function of time under various applied FC dosages.

The hydrodynamic diameter (HDD) of the precipitates versus reaction time was measured in pure water and Sb(III, V) suspensions at four different molar concentrations of FC coagulant (Figure 3 and Figure S3). The effect of Sb oxyanions on HDD of Fe precipitates was found to be insignificant. For instance, at 0.05 mM FC dosage, the size of precipitates was observed in the range of 1313 nm to 1357 nm for 23 min in all suspensions. Even at higher FC dosages of 0.10 mM, 0.15 mM and 0.20 mM, the size of Fe precipitates was found in the range of 3269 nm to 3637 nm, 5682 nm to 5915 nm and 8045 nm to 8365 nm, respectively (Figure 3A,B and Figure S3). The slightly larger sized Fe precipitates formed in Sb(V) suspensions may be attributed to the interaction between Sb(OH)_6_^−^ and Fe(OH)_2_^+^ species (Figure 3, Figures S1C,D and S3). This will lower the zeta potential of flocs close to pH_iep_, resulting in effective compression of an electrical double layer leading to a rapid aggregation process [14,22]. Notably, the significant increase in Fe floc size was observed upon increasing FC dosage (Figure 3A,B). Such an increase in the aggregation rate may be related to the following: (1) the increase in concentration of early-formed precipitates, (2) a higher supersaturation would reduce the interacting energy barriers between the primary precipitates, and (3) an increase in collision efficiency between precipitates, thus leading to promotion of precipitate aggregation rate [23,24]. In summary, the rapid formation of greater sized precipitates at higher coagulant concentration during sweep coagulation phase is critical in water treatment applications.

### 2.4. Influence of Contaminant Loading on Fe Precipitates Properties and Sb Coagulation

**Figure 4:**
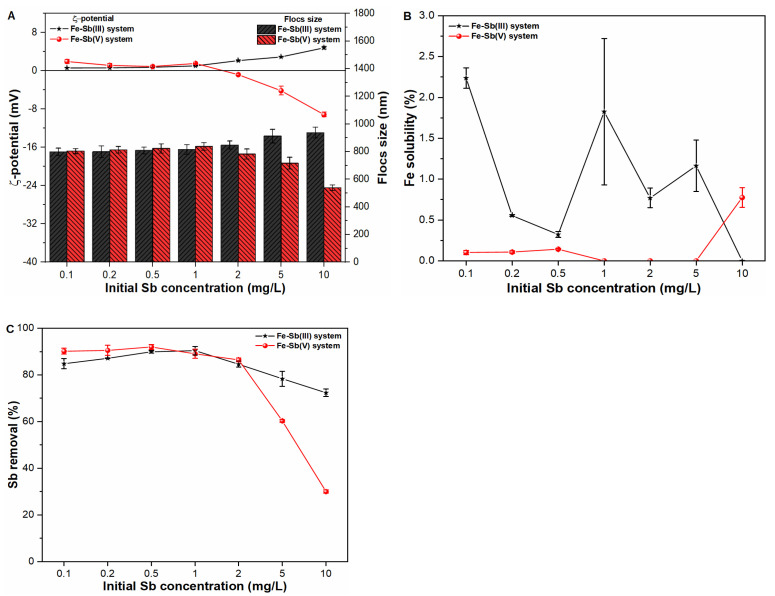
Influence of contaminant loading on (**A**) zeta potential (mV) and size (nm) of freshly formed Fe flocs; (**B**) Fe solubility (%) and; (**C**) Sb removal (%) in Sb(III, V) suspensions.

The experiments were also conducted to explore the influence of contaminant loading on zeta potential and size of primary flocs (Figure 4A). The presence of higher quantity of Sb(V) ions (above 2 mg/L) changes the ζ-potential values towards negative trajectory, with the highest influence at 10 mg/L Sb(V) suspension. The ζ-potential values at 2, 5 and 10 mg/L Sb(V) suspensions were observed to be −0.78 mV, −4.15 mV and −9.15 mV, respectively. Such differences may be attributable to the addition of more Sb(OH)_6_^−^ species in suspension (Figure S1D), which provide the pathways of electron transferring to surficial Fe atom, thus resulting in more negative zeta potential on Fe precipitates [14]. Similarly, higher concentration of Sb(V) ions had a detrimental effect on the size of primary flocs, with much lower value of 539.18 nm at 10 mg/L Sb(V) loading (Figure 4A). In contrast, positive ζ-potential value (4.826 mV) and greater sized (937.85 nm) Fe precipitates were observed in a suspension containing a higher concentration, i.e., 10 mg/L of Sb(III) ions. Such behavior may be attributable to the chemical bonding of Sb(OH)_3_ species with Fe(OH)_2_^+^ thus indicating strong interactive behavior leading to greater sized floc formation during the sweep coagulation phase [9,22].

After investigating the physicochemical properties of primary precipitates, the Fe solubility behavior was also examined, as shown in Figure 4B. The insignificant influence of both Sb oxyanions on Fe precipitation was observed in all suspensions, thus indicating sufficient availability of active Fe surface sites for complexation with both toxicants. Even so, the removal efficiency of pentavalent Sb ions significantly decreases to 60.3% and 30.04% at higher Sb(V) loading of 5 and 10 mg/L, respectively (Figure 4C). Such distinctive behavior may be ascribed to the role of electrostatic repulsive forces induced by a greater quantity of Sb(OH)_6_^−^ species and FeOSbO(OH)_4_^−^ complexes in such suspensions [9,25,26]. In addition, the negative ζ-potential values of −4.15 mV and −9.15 mV on Fe-Sb(V) complex also resulted in enhancing the mobility of Sb(OH)_6_^−^ species in such suspensions (Figure 4A,C). In contrast, consistent removal behavior of Sb(III) ions was observed, even in suspensions containing higher contaminant concentration. These results suggested that trivalent Sb ions can be effectively coagulated with Fe(III) ions irrespective of contaminant loading during conventional treatment process. Similarly, previous studies [9,17] also presented higher adsorption affinity of Sb(III) species towards various iron-based coagulants during water treatment.

### 2.5. Influence of Phosphates on Fe Precipitates Properties and Sb Coagulation

**Figure 5:**
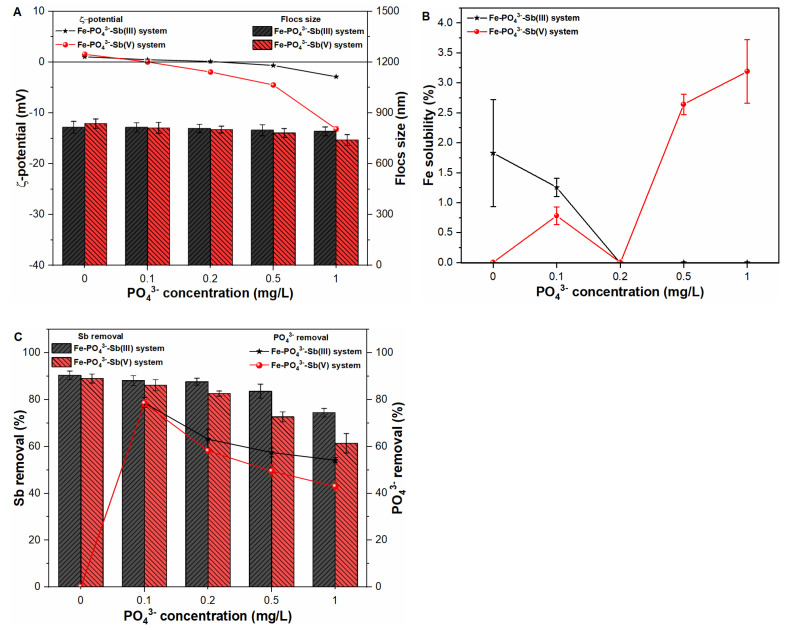
Influence of PO_4_^3−^ concentration on (**A**) zeta potential (mV) and size (nm) of freshly formed Fe flocs; (**B**) Fe solubility (%) and; (**C**) Sb removal (%) and PO_4_^3−^ removal (%) in Sb(III, V) suspensions.

The presence of anions, i.e., PO_4_^3−^ may alter the physicochemical characteristics of primary flocs, thereby affecting the overall removal process. Therefore, the ζ-potential and size of primary precipitates were monitored by adding 0.1 to 1 mg/L PO_4_^3−^ in pure water and Sb suspensions (Figure 5A and Figure S4A). The significant shift in ζ-potential values of precipitates from 1.56 mV to −13.15 mV was observed under coexistence of PO_4_^3−^ ions in Sb(V) suspensions (Figure 5A). Such observation may be supported by the fact that PO_4_^3−^ ions form has strong affinity towards Fe precipitates, thus ultimately affecting zeta potential of precipitates [9,13]. However, a negligible impact on the size of Fe precipitates was observed in all studied waters, which is consistent with earlier study [22], which reported that PO_4_^3−^ ions produce larger size precipitates after interacting with aluminum salts during coagulation process. In comparison, the Fe-PO_4_^3−^-Sb(III) system presented negligible impact on zeta potential and size of primary flocs (Figure 5A). The Fe solubility behavior was also observed in both the Fe-PO_4_^3−^-Sb(III) and Fe-PO_4_^3−^-Sb(V) systems, which indicated a negligible influence on Fe precipitation in all studied waters (Figure 5B).

Figure 5C presents the coagulation behavior of both Sb oxyanions and PO_4_^3−^ species under varying PO_4_^3−^ concentration. The results showed that the removal performance of the system decreases with increasing PO_4_^3−^ concentration from 0.1 mg/L to 1 mg/L. The much greater impact on Sb(V) removal may be ascribed to the strong competition between anionic Sb(V) species and anionic PO_4_^3−^ ions for Fe(III) sorption sites [27]. Furthermore, PO_4_^3−^ ions also compete with neutral Sb(III) ions owing to the fact that Sb and P are elements of group V with the same s2p3 configuration in the valence shell [13]. Thus, our results indicated decrease in Sb(III) removal upon increasing PO_4_^3−^ concentration in the suspension (Figure 5C). In order to further explore the affinity of PO_4_^3−^ ions towards Fe sorption sites, PO_4_^3−^ removal was also monitored in pure water as well as Sb suspensions (Figure 5C and Figure S4B). As shown in Figure S4B, the removal of PO_4_^3−^ ions decreased from 87.18% to 72.96% in pure water with initial anion concentration range of 0.1mg/L to 10 mg/L. Under coexisting environment, PO_4_^3−^ removal was observed in the range of 78.68% to 54.15% and 78.48% to 42.97% in Sb(III) and Sb(V) suspensions, respectively (Figure 5C). Such an inhibitory effect on Sb as well as PO_4_^3−^ removal indicates strong competition of both elements for available Fe sites. Therefore, the influence of anions is one of the main considerable factors, when removing heavy metals from drinking water.

### 2.6. Influence of Organics on Fe Precipitates Properties and Sb Coagulation

In order to further explore the effect of organic substances on the characteristics of Fe flocs and Sb coagulation from water, coagulation experiments were conducted using hydrophobic as well as hydrophilic organic ligands. The below sub sections will provide the comprehensive details on the Fe precipitates properties as well as on the interaction of Fe species with Sb contaminants in different aqueous matrices containing humic and salicylic acid.

#### 2.6.1. Humic Acid

**Figure 6:**
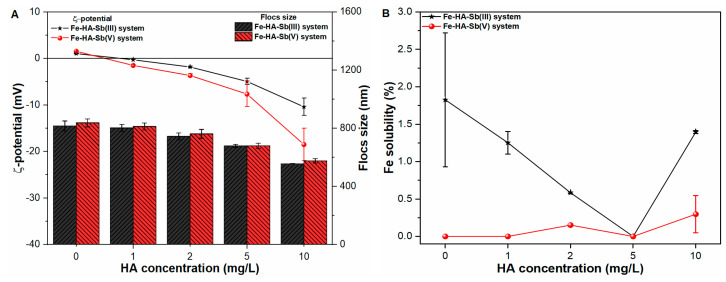

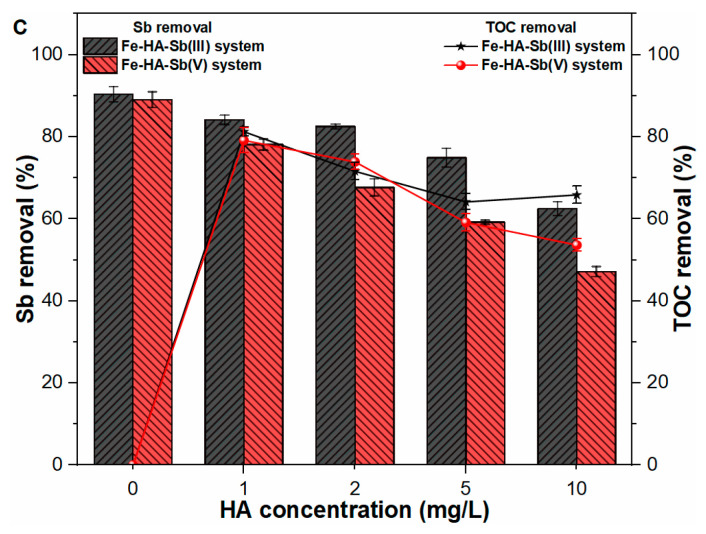
Influence of HA concentration on (**A**) zeta potential (mV) and size (nm) of freshly formed Fe flocs; (**B**) Fe solubility (%) and; (**C**) Sb removal (%) and TOC removal (%) in Sb(III, V) suspensions.

Natural organic matter such as humic acid (HA) ubiquitously exist in natural water environment. Therefore, the influence of HA on the zeta potential and size of primary Fe flocs were analyzed in pure water and Sb suspensions (Figure S5A and Figure 6A). The results indicated decrease in zeta potential and flocs size upon increasing HA concentration in all studied water samples. However, the coexistence of higher concentration of HA molecules (10 mg/L) in Sb(V) suspensions significantly lower the ζ-potential values to −18.49 mV, when compared with −10.39 mV and −11.15 mV in Sb(III) suspension and pure water, respectively. Such results are in good agreement with a strong interaction of anionic Sb(OH)_6_^−^ and HA molecules with Fe(III) complexes under a coexisting environment [17]. Moreover, the strong binding features between Sb(V) ions and HA molecules may also synergistically reverse the zeta potential of Fe precipitates to more negative trajectory in such suspensions [28,29,30]. This in turn was responsible for remarkably decreasing the flocs size from 837.19 nm to 576.10 nm in the Fe-HA-Sb(V) system. Similarly, the floc size also followed a similar decreasing trend from (815.61 nm to 556.19 nm) and (822.16 nm to 592.75 nm) in Sb(III) suspension and pure water, respectively. Such a significant decrease in the size of primary precipitates may be related to the strong binding affinity of high molecular weight anionic HA complexes with cationic Fe species in such suspensions [31,32]. In accordance, Fe stability was also monitored in Sb(III, V) suspensions, with results indicating insignificant impact on Fe precipitation in all studied waters (Figure 6B).

Figure 6C indicates the coagulation performance of both Sb oxyanions and TOC removal under varying HA concentrations. The results indicated that the removal performance of the system decreases upon increasing HA concentration from 0 mg/L to 10 mg/L. The significant decrease in Sb removal may be ascribed to the strong competitive inhibition by HA molecules (as indicated by % TOC removal in Figure 6C). Moreover, the HA molecules contain reactive groups namely hydroxyl, phenolic and amine, etc., with a strong complexation affinity towards Fe(III) sorption sites [33,34,35,36]. In addition, previous studies [32,36] also indicated lower trivalent and pentavalent Sb coagulation potential in the presence of humic substances owing to the fact that HA can bind with Sb oxyanions, hindering its adsorption onto precipitate surface sites, and thereby, enhancing the mobility of both contaminants in solution. In order to further examine the sorption affinity of HA molecules towards Fe sorption sites, TOC removal was also monitored in pure water as well as Sb suspensions (Figure 6C and Figure S5B). As shown in Figure S5B, the TOC removal decreases from 93.51% to 84.18% in pure water with HA concentration ranging from 1 mg/L to 10 mg/L. Under coexisting conditions, TOC removal was observed in the range of 81.26% to 65.87% and 79.15% to 53.65% in trivalent and pentavalent Sb suspensions respectively (Figure 6C). Such an antagonistic effect on Sb as well as TOC removal indicate strong competition of both elements for available Fe sites. These findings suggested that the presence of hydrophobic organic matter in water bodies could have a significant impact on the systems’ coagulation performance, particularly when removing heavy metal ions from drinking water reservoirs.

#### 2.6.2. Salicylic Acid

**Figure 7:**
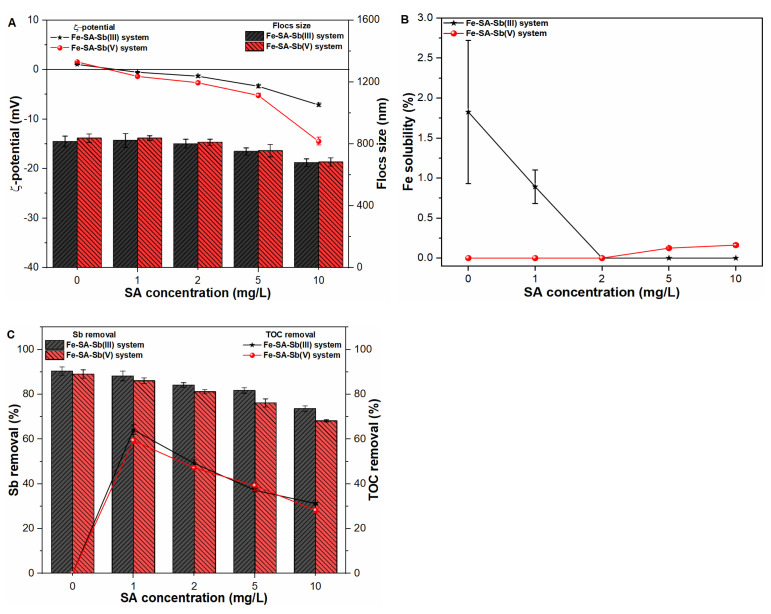
Influence of SA concentration on (**A**) zeta potential (mV) and size (nm) of freshly formed Fe flocs; (**B**) Fe solubility (%) and; (**C**) Sb removal (%) and TOC removal (%) in Sb(III, V) suspensions.

In order to analyze the influence of hydrophilic organic matter, i.e., salicylic acid (SA) on the ζ-potential and size of Fe flocs, experiments were conducted under varying SA concentration in pure water and Sb test solutions (Figure S6A and Figure 7A). The results indicated a slight decline in ζ-potential values and flocs size upon increasing SA concentration in all studied waters. Similar to the case of HA molecules, the higher concentration of SA molecules (10 mg/L) also presented more negative ζ-potential (−14.48 mV) as compared to −7.13 mV and −5.93 mV Sb(III) suspension and pure water, respectively. Such an observation indicates the major role of negatively charged Sb(V) ions in altering the surface properties of Fe precipitates. Furthermore, the binding affinity of low molecular weight SA molecules towards Fe(III) precipitates was also found weaker than that of HA molecules (Figure 6C and Figure 7C). Similarly, our previous studies [32,37] also showed a weak binding affinity of SA molecules with Fe as well as Sb species due to the presence of weaker acidic groups. Therefore, Fe-SA-Sb systems presented less decline in flocs size as compared to Fe-HA-Sb systems (Figure 6A and Figure 7A). The sizes of primary precipitates at higher SA concentration, i.e., 10 mg/L were measured to be 687.15 nm, 679.19 nm and 682.96 nm in pure water, Sb(III) and Sb(V) suspensions, respectively (Figure 7A). Fe precipitation was also monitored in all suspensions with result indicating negligible influence of SA molecules on flocs formation, as shown in Figure 7B.

Figure 7C indicates the coagulation efficiency of both Sb oxyanions and TOC removal under SA concentration range 0 mg/L to 10 mg/L. It was observed that the removal efficiency of both contaminants decreases with an increase in SA concentration. Compared to HA molecules, a relatively lesser effect on system performance was noticed in the presence of SA molecules. Such behavior may be ascribed to the existence of weaker acidic group in SA molecules with weaker binding affinity with Fe precipitates, thus retaining less contaminants in suspension [32,37]. Even though, the presence of SA molecules still affected the overall treatment process owing to competitive inhibition. Therefore, TOC removal was also analyzed in pure water as well as Sb(III, V) suspensions under varying SA concentrations (Figure 7C and Figure S6B). As shown in Figure S6B, the TOC removal decreases from 87.98% to 63.50% in pure water with SA concentration ranging from 1 mg/L to 10 mg/L. Under a co-occurring environment, TOC removal decreased in the range of 64.18% to 39.19% and 59.33% to 28.16% in trivalent and pentavalent Sb suspensions, respectively (Figure 7C). These results suggested that hydrophilic organic matter such as SA molecules have smaller adsorption affinity towards Fe precipitates and thus pose less of an impact on Sb coagulation behavior. In general, our findings suggested that the nature of organic ligands influences the physicochemical properties of Fe precipitates, thereby affecting the coagulation performance of natural water systems.

## 3. Materials and Methods

### 3.1. Chemicals and Stock Solutions Preparation

The 0.1 M coagulant stock solution and 100 mg/L phosphate solution were prepared by adding ferric chloride hexahydrate [FeCl_3_·6H_2_O (FC)] and sodium dihydrogen phosphate [NaH_2_PO_4_·H_2_O] in pure water. The 100 mg/L stock solutions of Sb(V) and Sb(III) oxyanions were prepared by dissolving potassium hexahydro-antimonate [KSb(OH)_6_] and antimony (III) oxide [Sb_2_O_3_] in pure water and 2 M hydrochloric acid [HCl] solution, respectively. The 100 mg/L stock solutions of hydrophobic (HA) and hydrophilic (SA) organic matter were prepared by adding powder in pure water. The HA and SA powder chemicals were purchased from Sigma-Aldrich (St. Louis, MO, USA). The sodium hydroxide [NaOH] solution was used to adjust pH of HA stock solution to 11, stirred at 100 rpm for 24 h. Further details can be found elsewhere [38]. These model organic matters are extensively used in water relevant research activities to simulate a natural aqueous environment [35,39]. The synthetic test solutions were then prepared by spiking desired volume of sample in pure water. Before the coagulation experiments, the total organic carbon (TOC) content with 0–10 mg/L organic matter for HA (0–4.17 mgC/L) and for SA (0–2.4 mgC/L) were analyzed. The ultra-pure Milli-Q water system (Millipore Co., Bedford, MA, USA) was used to obtain pure water. All glassware was washed with 15% nitric acid [HNO_3_] followed by cleaning with pure water.

### 3.2. Experimental Design and Procedures

A series of coagulation experimental trials were conducted using a jar tester (Hana Tech, Young Hana Tech Co., Ltd., Bucheon, Gyeongsangbuk-Do, Korea) with six paddles in a 250-mL Pyrex glass beaker. Initially, the effect of acidic (5.0), neutral (7.0) and alkaline (9.0) pH on the physicochemical properties of early formed Fe precipitates was monitored using 0.1 mM FC coagulant in pure water and 1 mg/L Sb(III, V) suspensions. Afterwards, the Fe solubility behavior and Sb coagulation performance was analyzed. The similar strategy was applied in the follow up experiments by setting pH to 7.0. In the next phase, only FC dosage was varied between 0.05–0.2 mM and agglomeration of Fe precipitates was monitored for the time duration of 1–23 min for the individual set of experiments. The influence of varying Sb(III, V) (0.1–10 mg/L) concentrations were also investigated at 0.1 mM FC dosage. In order to further investigate the effect of anion (0–1 mg/L PO_4_) and organic matter (0–10 mg/L HA and SA), separate series of experiments were conducted at circumneutral pH using 0.1 mM FC dosage in both pure water and 1 mg/L Sb(III, V) suspensions. All the experiments were conducted in duplicate and relative standard deviation (RSD) were reported.

The operating parameters were set based on a typical coagulation–flocculation process used in water treatment plants. These include rapid coagulation phase (140 rpm for 3 min); flocculation phase (40 rpm for 20 min) and sedimentation phase (30 min) [9,17]. In all experiments, the predetermined dosage of FC coagulant was added into the test samples at the start of rapid mixing phase. The zeta potential and size of freshly formed Fe flocs were measured after 3 min coagulation phase for all experimental conditions except agglomeration experiments. The aliquots were collected after completion of the experiment and TOC was measured using unfiltered sample using TOC analyzer with an ASI-L liquid autosampler (Shimadzu Corp, Kyoto, Japan). While the residual Fe and Sb concentration were analyzed after filtering through 0.45 μm filter paper using inductively coupled plasma optical emission spectrometer (ICP-OES: Agilent Technologies, Sana Clara, CA, USA). The remaining phosphate was analyzed through the ammonium molybdate spectrophotometric method at a wavelength of 700 nm using ultraviolet-visible (UV) spectrophotometer (Optizen 2120 UV, Mecasys, Daejeon, Korea) as described elsewhere [40,41]. The Fe(III) and Sb(III, V) speciation diagrams were plotted using Visual MINTEQ 3.1. (KTH, Stockholm, Sweden).

The ζ-potential and size of Fe precipitates were analyzed using a Zetasizer (NanoZS, Malvern, Worcestershire, UK). The ζ-potential values were not measured directly rather electrophoretic mobility (u_E_) was first measured by measuring particle velocity (V_P_: μm/s) under some applied electric field (E_x_: Volt/cm) using following Equation (1).

(1) $$u_{E}=\frac{V_{P}}{E_{x}}$$

Afterwards, zeta potential was calculated from measured using the Henry equation (Equation (2)).

(2) $$u_{E}=\frac{2\varepsilon \zeta }{3\eta }\;.\;F\left(\kappa a\right)$$

where ε is permittivity of dielectric constant, ζ is zeta potential (mV), F(κa) is Henry correction factor and η is the liquid viscosity. Here, κ is the reciprocal double-layer thickness calculated based on the ionic strength of the coagulated system, a is the radius of the particles measured by the Zetasizer.

## 4. Conclusions

This research work investigated the characteristics of Fe flocs such as ζ-potential and size in pure water and Sb(III, V) suspensions under a heterogeneous aqueous environment. In addition, coagulation behavior of Sb oxyanions was also explored under similar conditions. Our results demonstrated that the zeta potential of precipitated Fe is a function of pH, where point of zero charge found near at neutral pH environment in all tested water samples. Furthermore, the results of flocculation experiments indicated similar surface characteristics of Fe flocs in all studied waters under a circumneutral pH environment. At pH 9, Sb(V) removal was remarkably decreased due to enhanced Fe solubility in such a system. The significant decline in floc size and charge reversal characteristics enhance the mobility of Sb(V) species in water at higher contaminant loading rate. The influence of anions such as phosphate substantially affect the surface characteristics of Fe flocs as well as the removal behavior of Sb oxyanions. The impact of hydrophobic (HA) and hydrophilic (SA) organic compounds on surface properties of precipitated Fe was observed, with greater variation in the presence of HA molecules, thereby affecting the Sb removal efficiency. In general, these findings suggested that the physicochemical characteristics of Fe precipitates are governed by solution chemistry, thereby may affect the overall removal performance of heavy metal ions from drinking water.

## Data Availability

All data used to support the findings of this study are included within the article.

## Supplementary Materials

The following are available online. Figure S1: (A) Influence of pH on zeta potential (mV) and size (nm) of freshly formed Fe flocs; (B) Fe solubility (%) in pure water and; (C,D) Speciation diagrams of Fe(III) and Sb(III, V) drawn using Visual MINTEQ 3.1, Figure S2: Influence of FC dosages on zeta potential (mV) and size (nm) of freshly formed Fe flocs in pure water, Figure S3: Aggregation of Fe precipitates in pure water as a function of time under various applied FC dosages, Figure S4: Influence of PO_4_^3−^ concentration on (A) zeta potential (mV) and size (nm) of freshly formed Fe flocs and; (B) PO_4_^3−^ removal (%) in pure water, Figure S5: Influence of HA concentration on (A) zeta potential (mV) and size (nm) of freshly formed Fe flocs and; (B) TOC removal (%) in pure water, Figure S6: Influence of SA concentration on (A) zeta potential (mV) and size (nm) of freshly formed Fe flocs and; (B) TOC removal (%) in pure water.

## Sample Availability

Samples of the compounds are not available from the authors.
