# Supplementary material for: Coagulation Behavior of Antimony Oxyanions in Water: Influence of pH, Inorganic and Organic Matter on the Physicochemical Characteristics of Iron Precipitates
Source: Molecules. 2022 Mar 3;27(5):1663. doi: 10.3390/molecules27051663 (PMC8912098; doi:10.3390/molecules27051663)
Supplement: Supplementary file 1 [file molecules-27-01663-s001.zip › molecules-1573009-supplementary.pdf]

molecules

Supplementary Materials

Coagulation Behavior of Antimony Oxyanions in Water: Influence of pH, Inorganic and Organic Matter on the Physicochemical Characteristics of Iron Precipitates

Muhammad Ali Inam [1], Kang Hoon Lee [2,*], Hira Lal Soni [3], Kashif Hussain Mangi [3], Abdul Sami Channa [3], Rizwan Khan [3], Young Min Wie [4] and Ki Gang Lee [4]

[1] Institute of Environmental Sciences and Engineering (IESE), School of Civil and Environmental Engineering (SCEE), National University of Sciences and Technology (NUST), H-12 Campus, Islamabad 44000, Pakistan; ainam@iese.nust.edu.pk
[2] Department of Energy and Environmental Engineering, The Catholic University of Korea, 43 Jibong-ro, Bucheon-si, Gyeonggi-do 14662, Korea
[3] Department of Chemical Engineering, Quaid-e-Awam University of Engineering, Science and Technology (QUEST), Nawabshah 67480, Sindh, Pakistan; soni.hiralal@quest.edu.pk (H.L.S.), kashifmangi@quest.edu.pk (K.H.M.); abdul.sami@quest.edu.pk (A.S.C.); rizwansoomro@quest.edu.pk (R.K.)
[4] Department of Materials Engineering, Kyonggi University, Suwon 16227, Korea; supreme98@kyonggi.ac.kr (Y.M.W.), gglee@kyonggi.ac.kr (K.G.L.)
* Correspondence: diasyong@catholic.ac.kr

Citation: Inam, M.A.; Lee, K.H.; Soni, H.L.; Mangi, K.H.; Channa, A.S.; Khan, R.; Wie, Y.M.; Lee, K.G. Coagulation Behavior of Antimony Oxyanions in Water: Influence of pH, Inorganic and Organic Matter on the Physicochemical Characteristics of Iron Precipitates. *Molecules* **2022**, *27*, 1663. https://doi.org/10.3390/molecules27051663

Academic Editors: Monika Wawrzkiewicz; Anna Wołowicz

Received: 11 January 2022
Accepted: 1 March 2022
Published: 3 March 2022

Publisher's Note: MDPI stays neutral with regard to jurisdictional claims in published maps and institutional affiliations.

Copyright: © 2022 by the authors. Submitted for possible open access publication under the terms and conditions of the Creative Commons Attribution (CC BY) license (https://creativecommons.org/licenses/by/4.0/).

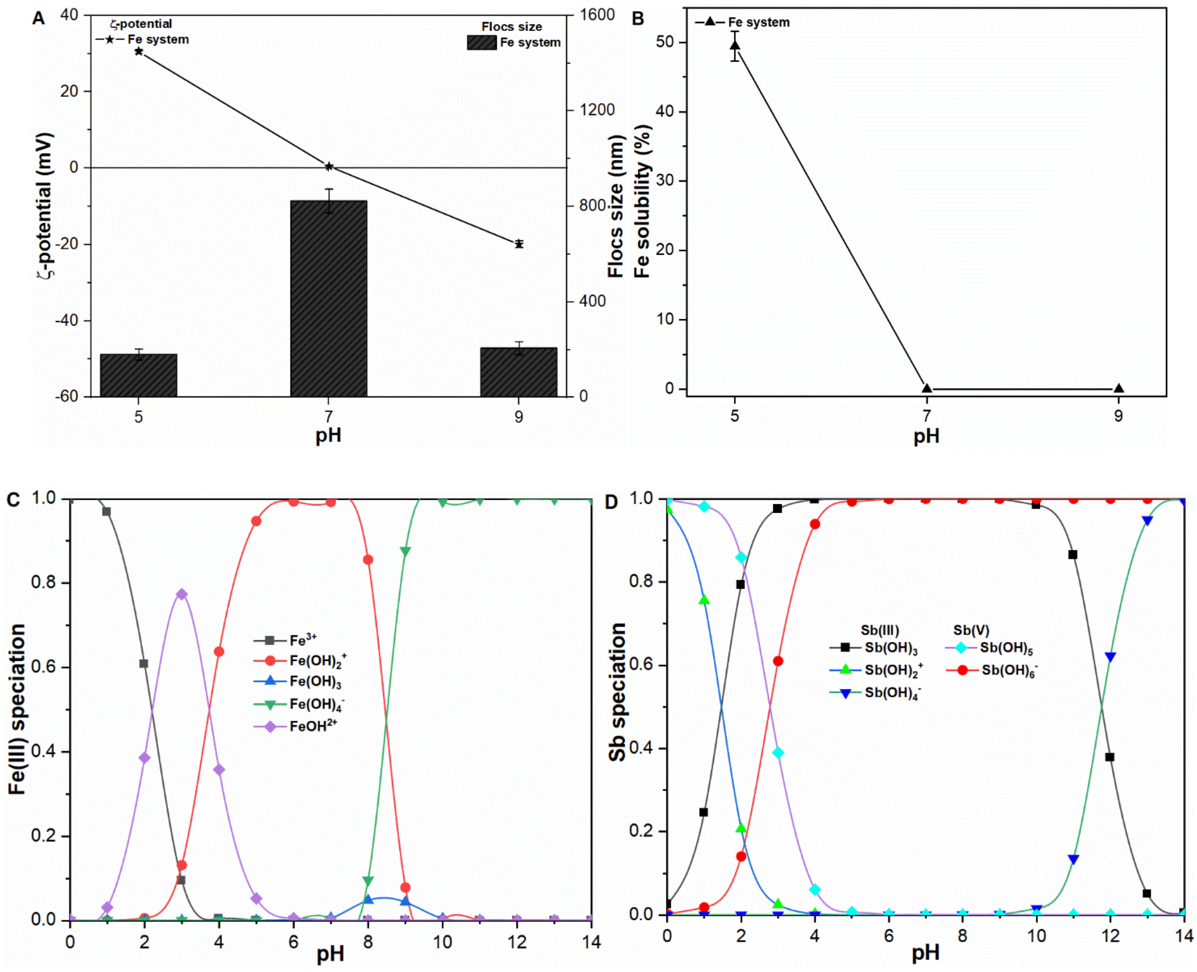

Figure S1: (**A**) Influence of pH on zeta potential (mV) and size (nm) of freshly formed Fe flocs; (**B**) Fe solubility (%) in pure water and; (**C**,**D**) Speciation diagrams of Fe(III) and Sb(III, V) drawn using Visual MINTEQ 3.1.

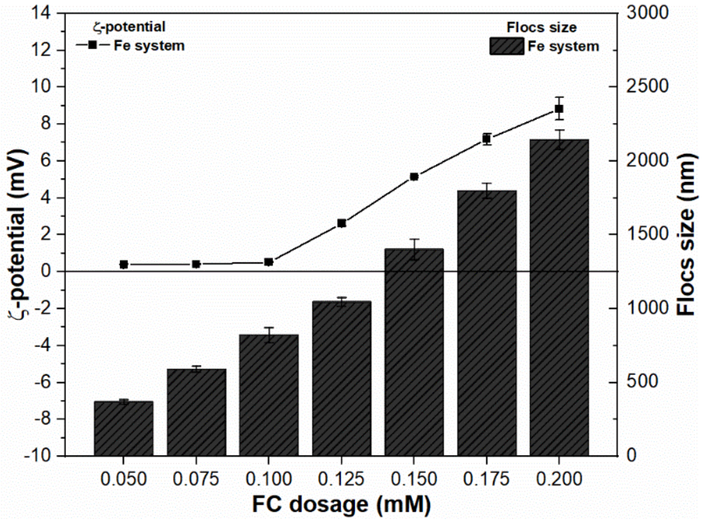

Figure S2: Influence of FC dosages on zeta potential (mV) and size (nm) of freshly formed Fe flocs in pure water.

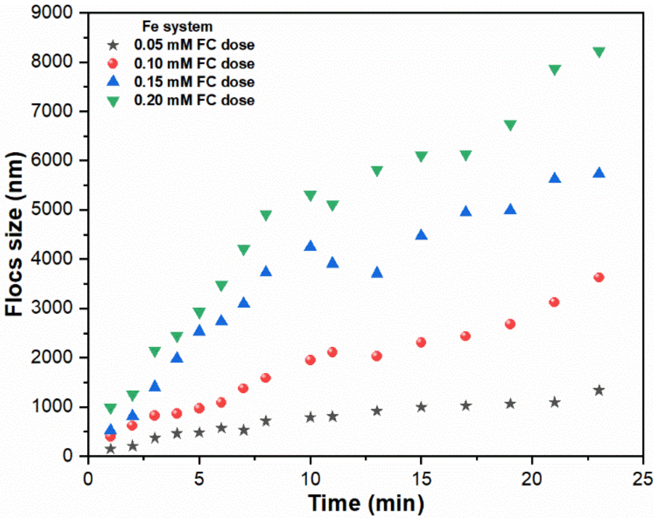

Figure S3: Aggregation of Fe precipitates in pure water as a function of time under various applied FC dosages.

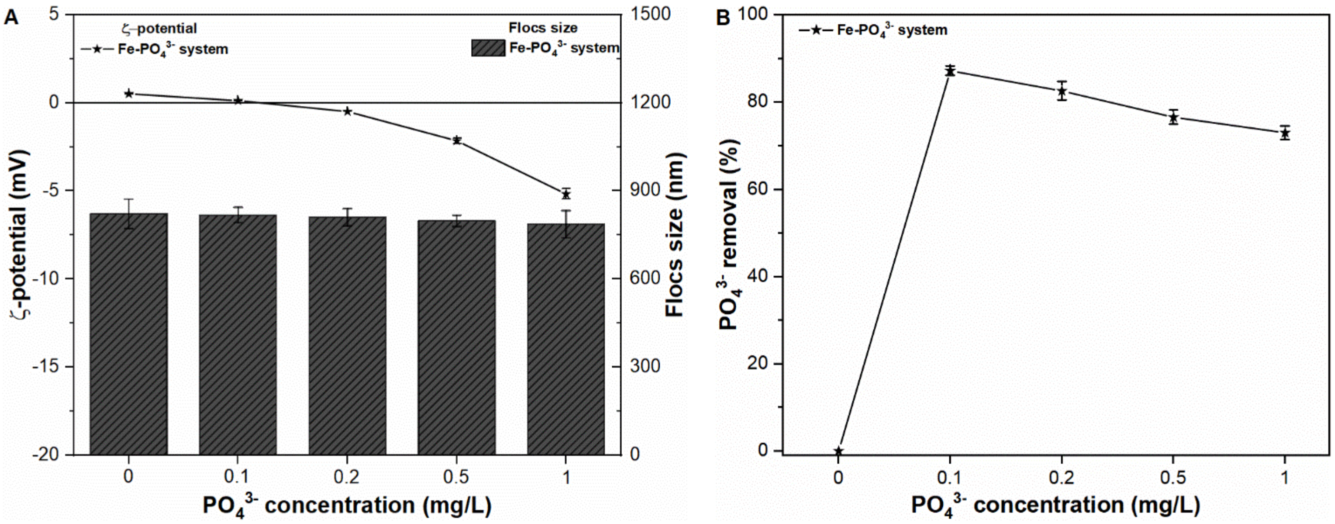

Figure S4: Influence of PO_4^{3-} concentration on (**A**) zeta potential (mV) and size (nm) of freshly formed Fe flocs and; (**B**) PO_4^{3-} removal (%) in pure water.

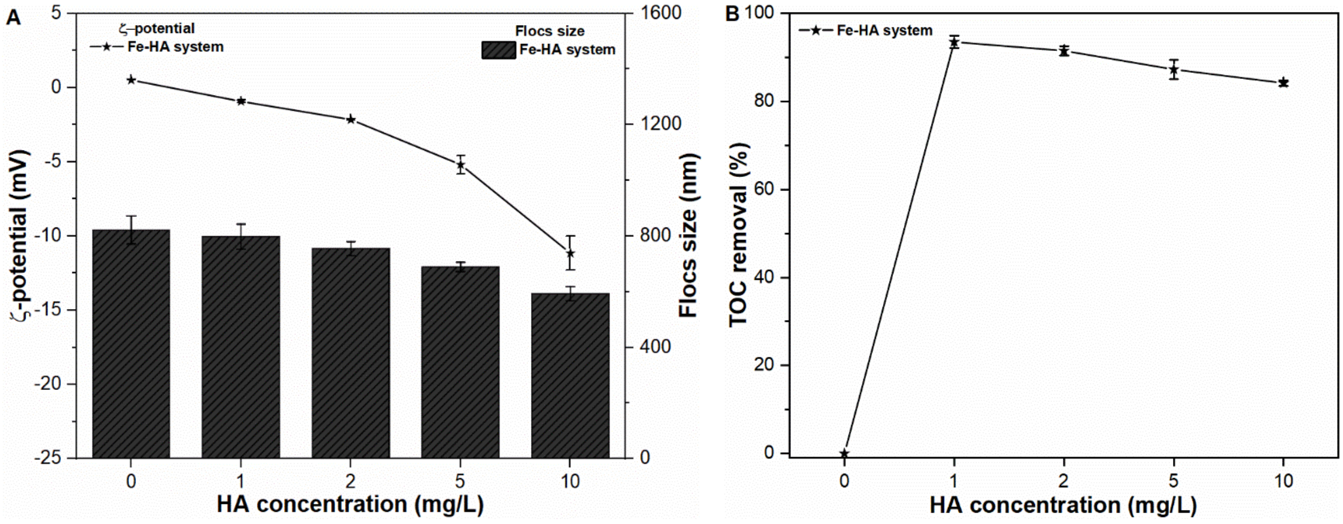

Figure S5: Influence of HA concentration on (**A**) zeta potential (mV) and size (nm) of freshly formed Fe flocs and; (**B**) TOC removal (%) in pure water.

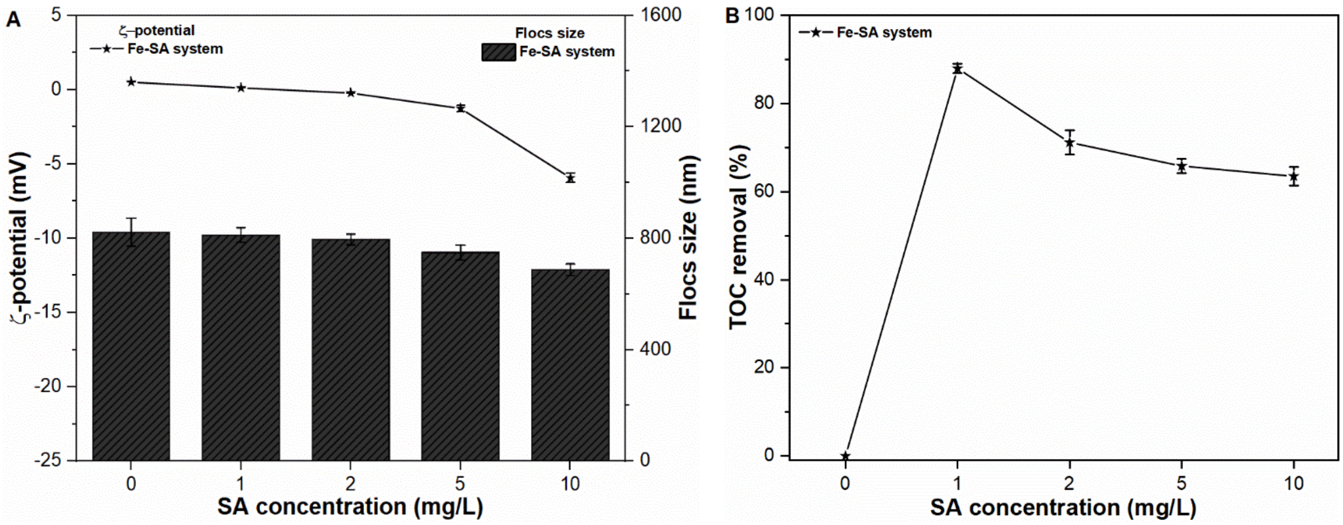

Figure S6: Influence of SA concentration on (**A**) zeta potential (mV) and size (nm) of freshly formed Fe flocs and; (**B**) TOC removal (%) in pure water.
